# Integrated metabolomic and transcriptome analyses reveal finishing forage affects metabolic pathways related to beef quality and animal welfare

**DOI:** 10.1038/srep25948

**Published:** 2016-05-17

**Authors:** José A. Carrillo, Yanghua He, Yaokun Li, Jianan Liu, Richard A. Erdman, Tad S. Sonstegard, Jiuzhou Song

**Affiliations:** 1 Department of Animal & Avian Sciences, University of Maryland, College Park, MD 20742, USA; 2 College of Animal Science and Technology, Northwest A&F University, Yangling, Shaanxi, P.R. China, 712100; 3 Recombinetics Inc., 1246 University Ave. W, St. Paul, MN 55104, USA

## Abstract

Beef represents a major dietary component and source of protein in many countries. With an increasing demand for beef, the industry is currently undergoing changes towards naturally produced beef. However, the true differences between the feeding systems, especially the biochemical and nutritional aspects, are still unclear. Using transcriptome and metabolome profiles, we identified biological pathways related to the differences between grass- and grain-fed Angus steers. In the *latissimus dorsi* muscle, we have recognized 241 differentially expressed genes (FDR < 0.1). The metabolome examinations of muscle and blood revealed 163 and 179 altered compounds in each tissue (P < 0.05), respectively. Accordingly, alterations in glucose metabolism, divergences in free fatty acids and carnitine conjugated lipid levels, and altered *β*-oxidation have been observed. The anti-inflammatory n3 polyunsaturated fatty acids are enriched in grass finished beef, while higher levels of n6 PUFAs in grain finished animals may promote inflammation and oxidative stress. Furthermore, grass-fed animals produce tender beef with lower total fat and a higher omega3/omega6 ratio than grain-fed ones, which could potentially benefit consumer health. Most importantly, blood cortisol levels strongly indicate that grass-fed animals may experience less stress than the grain-fed individuals. These results will provide deeper insights into the merits and mechanisms of muscle development.

In many parts of the world, beef constitutes a main dietary component and a major source of protein. Beyond amino acids, beef also provides abundant energy and essential vitamins and minerals such as iron, which is frequently deficient in the human diet^1^,^2^. The beef industry in developed countries is currently undergoing changes and is facing increased demands especially for natural meat^3^. This transformation is partly driven by the public concern regarding the environmental impacts of intensive livestock production, animal welfare, meat quality, and the flavor of beef^4^,^5^. Recently, consumers have preferred naturally-raised organic beef because they consider it to be safer and higher quality as this type of beef is obtained without using antibiotics or extrinsic hormones. Compared to its grain-fed counterpart, grass-fed beef is lower in total fat content and richer in omega-3 fatty acids, characteristics which could be more beneficial for human health^4^,^6^. As more humane husbandry becomes an integral part of quality assurance programs for consumer-accepted sustainable animal production^7^, such changes will also affect other production components like animal health, breeding, production management, downstream processing, meat quality, nutrient value and environmental impact.

As we know that animal growth, health, and meat quality are controlled by genetic and environmental factors. Considering diet regimen as a nutritional welfare variable, it influences the animal as a whole entity, affecting nutrient assimilation, disease resistance and behavior. Consumers often postulate that raising animals entirely on an all forage diet might be healthier for the animals as it is closer to the environment in which they have evolved. The general public understands that grain finished systems seem unnatural^2^. Although many reports claim that grass finishing is healthier and better for the environment, there is conflicting information regarding the benefits of grass-fed beef 8,9,10. Unfortunately, we found very little research quantifying the effects of natural production on fatty acid profiles, meat quality and animal welfare. On animal production, there is a severe lack of solid evidence that beef from grass-fed animals have these advantages, which further prevents animal producers interested in meeting the demand for grass finished beef from making fully informed management decisions.

Beef quality and behavior traits also share the property of being concurrently regulated by gene sets. The identification of genes that control critical beef quality traits, such as tenderness, fatty acid content, and marbling will help to unveil the regulatory networks and physiological mechanisms behind these clusters. Several reports suggest that gene expression would be mediated by environmental factors at the transcriptional and translational levels in different species^11^,^12^. For example, dietary polyunsaturated fatty acids (PUFA’s) dramatically affect the expression of genes, regulating four families of transcription factors: sterol regulatory element binding proteins (SREBPs) 1 and 2, liver X receptors (LXRs), hepatic nuclear factor-4 (HNF-4) alpha and peroxisome proliferator activated receptor (PPAR) alpha, beta and gamma^13^. These transcription factors play major roles in fatty acid, cholesterol, triglyceride, hepatic carbohydrate, and bile acid metabolism which demonstrates a complex interaction between diet, genotype and gene activity, and the diverse mechanisms of action of dietary small bioactive components^14^,^15^,^16^. The major approaches in metabolomic studies include mass spectrometry- (MS) and nuclear magnetic resonance (NMR)-based techniques. Detection of metabolites using MS-based techniques comprises several methods including gas chromatography (GC), high performance liquid chromatography (HPLC), and capillary electrophoresis (CE). The high throughput metabolomics technologies allow for the assessment of global low-molecular-weight metabolites and show great potential in biomarker discovery^17^,^18^. Such studies will definitely help to identify biologically meaningful metabolic networks that control cellular responses to environmental factors, including diet^19^,^20^. To date, few studies have investigated the nutritional regulation of the whole metabolome; therefore, the characterization of metabolite levels in beef should provide a unique opportunity to ascertain ‘metabolic signatures’ or ‘molecular hubs’ as biomarkers related to beef quality and different metabolomics platforms.

Metabolomic studies systematically assess the unique chemical elements produced during specific cellular processes by describing the metabolites profiles of individuals that undergo similar biological processes and transformations^21^. The fingerprint of a product could be characterized by its metabolites content^22^. The quality or origin of a food is basically determined by its biochemical components, which also will define the metabolites profile. RNA-Sequencing (RNA-Seq) analysis interrogates the level of mRNA from an organism to estimate its gene expression profile at a given moment. Identification of active and repressed genes in different regimens would lead to the discovery of the biological pathways affected by these functional differences. Accordingly, the aim of this study is to characterize molecular signatures from cDNA expression and metabolomics in blood and muscle originated from grass-fed and grain-fed steers to ascertain pathways and functional differences and to identify the differences in meat quality with implications for public health and animal welfare. Additionally, the discovery of involved pathways would benefit the meat industry in producing healthier and higher quality products.

## Results

### Growth curve and beef tenderness with diet styles

**Figure 1:**
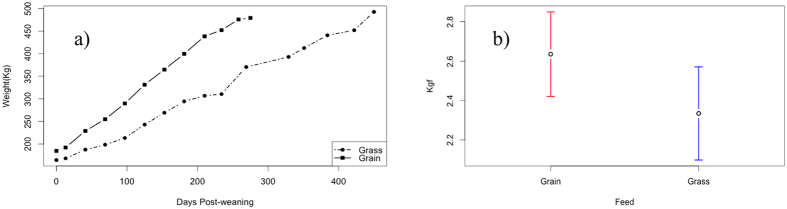
Growth curve and beef tenderness in different diets. (**a**) Body weights trajectories in grass-fed and grain-fed steers from weaning until termination (Grass, Grain: n = 30 and 36, respectively). (**b**) Warner-Bratzler Shear Force expressed in kilogram-force (kgf) in muscle obtained from grain-fed and grass-fed animals.

The growth curve of grass- and grain-fed Angus steers is shown in Fig. 1a. As expected from the difference in nutrients (mainly energy) provided by the feeding regimens, rate of weight gain differed between groups, the average daily gain (ADG) from weaning to termination was linear in both groups, but certainly displayed different slopes as grass-fed animals required approximately 200 additional days to reach minimum acceptable slaughter weight. The average final weights were 479.04 and 492.40 kg for the grain-fed and grass-fed categories (*p* > *0.05*). According to the Warner-Braztler Shear Force (WBS), diet type had no significant impact on beef tenderness (*p* > *0.05*) (Fig. 1b).

### Animal welfare and feed regimen

**Figure 2:**
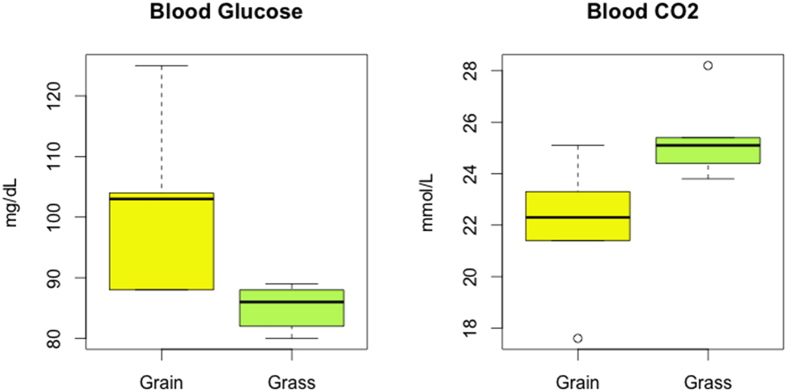
Glucose and CO2 blood levels in grain- and grass-fed steers. (**a**) Blood glucose level obtained through conventional clinical analysis, represented in milligram per deciliter (mg/dL) (n = 10 for each group); (**b**) Carbon dioxide measured in blood and expressed as milimol per liter (mmol/L) (n = 10 for each group).

**Figure 3:**
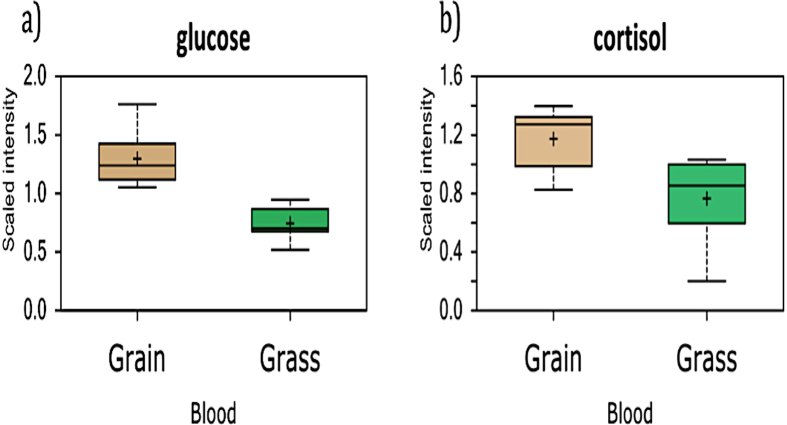
Glucose and cortisol metabolomics blood levels in grain and grass fed animals. (**a**) Glucose level measured in blood employing metabolomic approach (y axis represents mass spectrometry scaled intensity, n = 8 for each group). (**b**) Relative mass spectrometry scaled intensities of cortisol determined in blood of grass and grain fed steers (n = 8 for each group).

Initially, blood samples from animals receiving grass or grain diet were analyzed using standard hematological parameters, plus carbon dioxide and glucose content (Supplementary Table S1). We found that most parameters were similar in both groups except for carbon dioxide and glucose (Fig. 2). However, the metabolome examination revealed that glucose and cortisol levels in blood differed significantly between animals from the two feeding systems (Fig. 3a,b).

### Fatty acid content of beef and diet style

**Figure 4:**
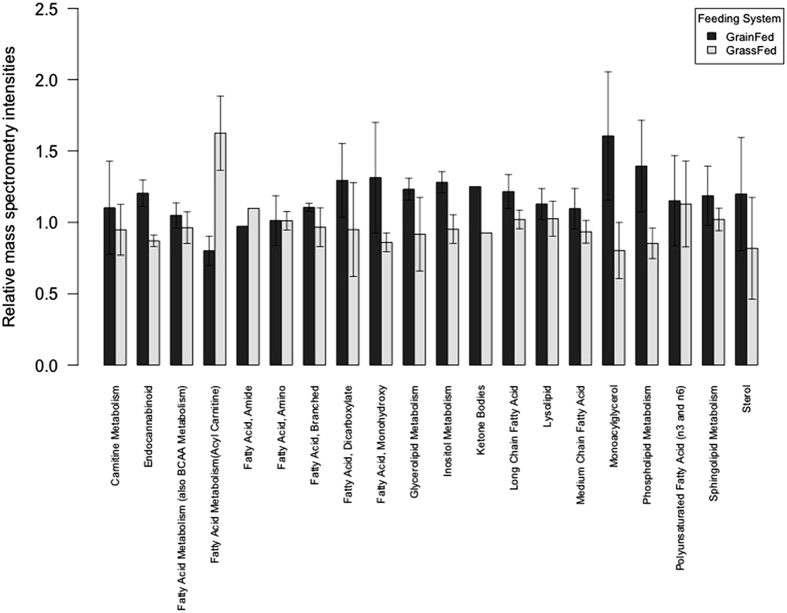
Averaged relative mass spectrometry intensities of 131 lipid molecules detected in muscle of grain-fed and grass-fed animals. There are 20 sub-pathways according to its physical and/or functional properties. Sub-pathways Fatty Acid, Amide and Ketone Bodies only have one member thus error bars are absent in these two groups.

A variety of fatty acids are found in muscle but for its relevance in human health, we examined the polyunsaturated fatty acids (PUFA) concentration of beef (Table 1). Eleven members of this group were recognized and showed significant variation between groups of animals that received either grass or grain, suggesting that type of feed might influence the final concentration of PUFAs in beef. Fold change was calculated as grain/grass mean, implying that values <1 represent more abundance of a biochemical in grass; contrarily, values >1 mean higher compound concentration in grain samples. Interestingly, all omega 3, eicosapentaenoate, docosapentaenoate and docosahexaenoate, were significantly higher in grass-fed beef. The opposite was observed for linoleate, dihomo-linolenate, arachidonate, adrenate, docosapentaenoate, and dihomo-linoleate, which happen to be omega 6 fatty acids. The metabolomic analysis detected 131 lipid molecules that have been summarized in 20 sub pathways according to their properties and functions. Figure 4 shows that most groups–excepting fatty acid metabolism (acyl carnitine) and fatty acid, amide–averaged higher lipid intensity in muscle from grain fed individuals, suggesting greater total fat concentration in this type of beef based on the subset of total lipid population measured with this metabolomic approach.

### Metabolomics Analysis

**Figure 5:**
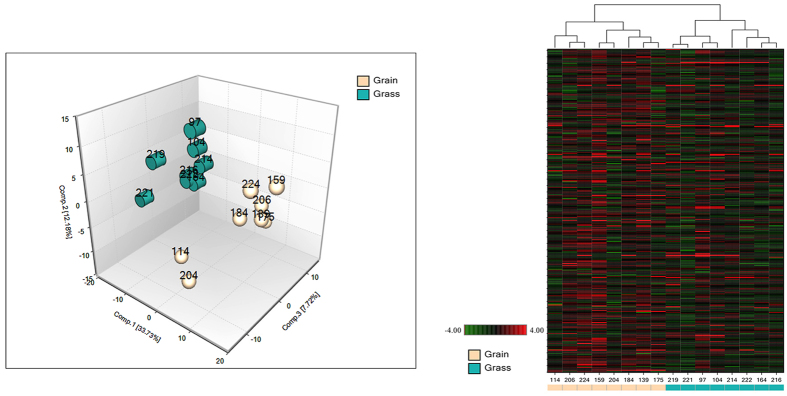
Principal Component Analysis (PCA) and hierarchical clustering obtained from employing metabolic concentration of all metabolites detected in muscle tissue. Turquoise and light-brown color represent grass and grain-fed groups, respectively. In the heatmap red color denotes enrichment of the corresponding molecules, contrary to green color that represents depletion (n = 8 for each group).

**Figure 6:**
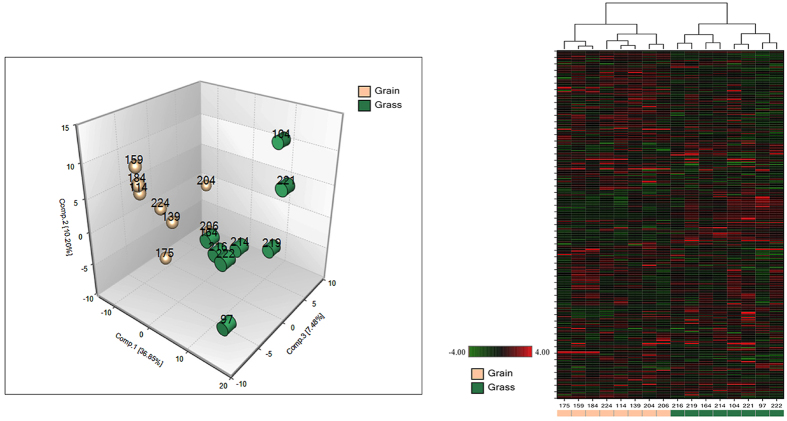
Principal Component Analysis (PCA) and heatmap acquired from metabolites relative expression calculated in blood of grain- and grass-fed steers. In the hierarchical clustering red color shows higher level of the corresponding biochemical, opposite to green that means reduction. Light brown and green colors represent grain and grass-fed individuals in both graphs (n = 8 for each group).

**Figure 7:**
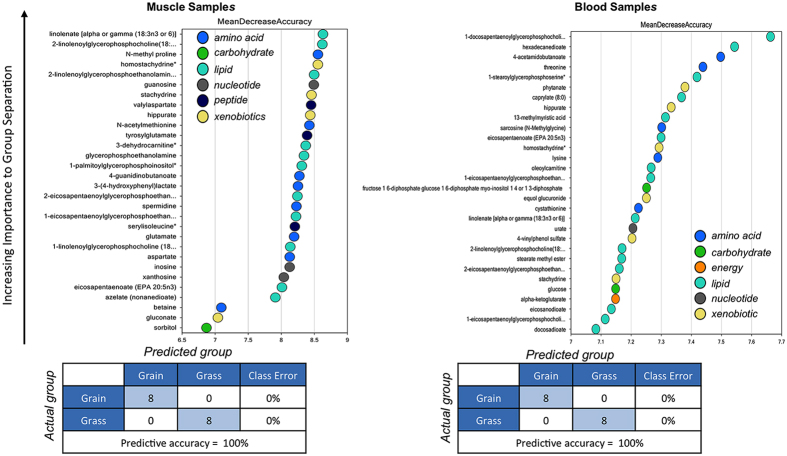
Random forest analyses obtained from muscle and blood. Random forest analysis consists in a supervised classification technique based on an ensemble of decision trees that has proven to be a valuable statistical tool for identifying biomarkers of interest. Random forest analysis based on the biochemicals detected in this dataset resulted in a predictive accuracy of 100% between dietary conditions. This value is greater than random chance (50%), suggesting these metabolites may be of interest as biomarkers. The y-axis represents the molecules in order of importance for group classification, from top to bottom. The legend represents the type of biochemical and the table below each chart shows the prediction accuracy based on the random forest result (actual against predicted group).

We examined a total of 326 and 353 known compounds in blood and muscle, respectively. A summary of the numbers of biochemicals that achieved statistical significance (p ≤ 0.05), as well as those approaching significance (0.05 ≤ p ≤ 0.1) is shown in Table 2. Principal component analysis and hierarchical clustering revealed a perfect separation between grass and grain finished muscle tissue and blood that may be indicative of the divergences in global metabolism (Figs 5 and 6). These figures demonstrate that both tissues–blood and muscle–are valid to classify the origin of the animal regarding their level of metabolites, considering the 326 and 353 correspondingly. We know that metabolites are product of biochemical reactions thus differences in those components are manifestations of distinct physiological states. Both groups clustered tightly in muscle, demonstrating a smaller variation of metabolites levels than in blood. Additionally, random forest analysis based on the biochemicals detected in this dataset resulted in a predictive accuracy of 100% between dietary conditions. The results show that the calculated values are greater than random chance, suggesting that those biochemicals can be of interest as biomarkers for the specific types of diet. Figure 7 indicates the top 30 metabolites and their classifications, summarized by the legend color code. In both tissues the top two biochemicals are lipids, followed by an amino acid. It is well known that both elements are critical to define beef quality, a trait that heavily depends on marbling. Surprisingly and despite the different level of energy in the different diets, carbohydrates compounds barely appear once in muscle and twice in blood, respectively.

### Glucose and Cortisol level

The level of cortisol found in the blood metabolomic analysis coincided with the trend observed in the regular blood test (Fig. 3b). Our study showed that grass-fed animals had lower cortisol levels, suggesting that animals maintained on pastures, following their natural habits, displayed less stress than animals maintained in restricted spaces with limited walking distances and high energy diets. The observed differences in blood glucose, CO_2_, and cortisol concentrations, reflect broad phenotypic traits that may be altered through many different mechanisms and these traits themselves could also affect other metabolic pathways. Thus, the result shows that grass-fed and grain-fed treatments generate broad metabolic divergences, providing an excellent opportunity to study interactions between nutrients and the genome.

### Gene Expression Analysis

To study the gene activity in both types of diet, mRNA from muscle was extracted to construct libraries for RNA-Seq analysis on Illumina Hi-Seq 2000. Each regimen was represented by two randomly chosen biological replicates, with a total number of 32,401,755 and 30,428,052 reads for grass-fed and grain-fed samples, respectively. The raw data has been deposited in NCBI database and can be access through this link: https://www.ncbi.nlm.nih.gov/geo/query/acc.cgi?acc=GSE70248. Mapping levels of the samples were adequate and similar, averaging 73% in the grain-fed group and 76% in the grass-fed cluster. Table 3 shows the mapping details of the samples.

**Figure 8:**
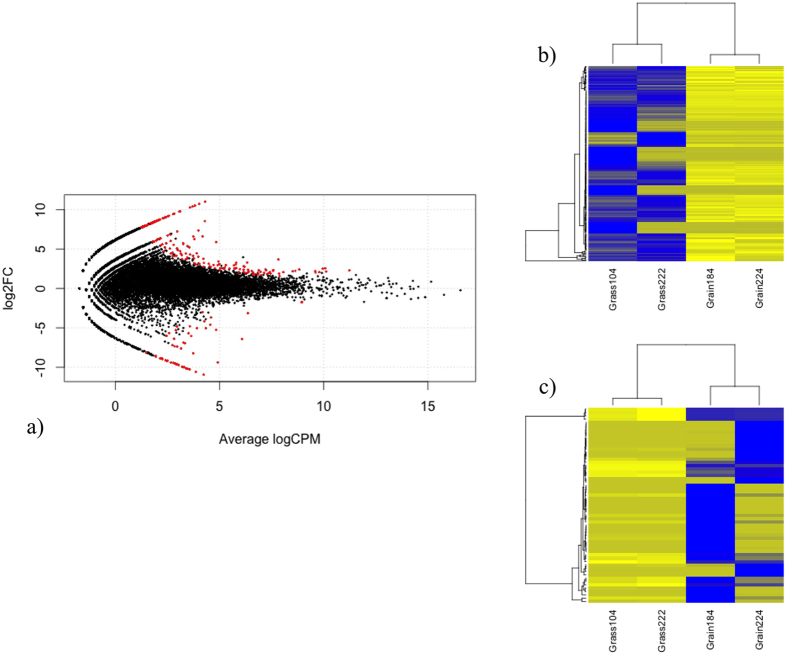
Differentially expresses genes. (**a**) MA plot shows the significant differentially expressed genes in red, (y-axis depicts the log2 fold change of gene expression between grass and grain fed groups; x-axis represents the average log reads count per million for each gene). (**b**) Hierarchical cluster of samples according to up-regulated genes in the grass-fed group. (**c**) Heatmap obtained using down-regulated genes in grass-fed individuals.

After analysis, we found 241 genes displaying differential expression (FDR < 0.1). From these genes, 59 and 182 demonstrated down- and up-regulation in grass-fed animals. The MA plot (M = log ratio and A = mean average) was used to illustrate the magnitude distribution of altered gene expression based on diet (Fig. 8a). The red dots represent the significant differentially expressed genes; the positive values on the y-axis are higher expressed in the grass-fed group however the negative ones showed a higher expression in grain-fed animals. Figure 8b shows the clustering of samples according to the expression level of up-regulated genes in the grass-fed group. The dendrogram showed that the expression of these 182 genes were more consistent within the grain-fed group. In the other hand, Fig. 8c illustrates the clustering of samples based on genes that were up regulated in grain-fed animals; in this case the variation of gene expression between replicates was larger for the grain-fed group. The lists of genes based on expression directionality are provided in the Supplementary Dataset S2 and Supplementary Dataset S3. The log2 fold-change levels ranged from −10.94 to 10.99, being almost symmetrical for both orientations. According to the FDR value, the next well-recognized genes *KRT5, GJA1, MDH1, CA12, CHST13, AMBP, GSTT3, NDUFA4* were identified among the top 10 entities.

### Pathway Analysis

The complete set of differentially expressed genes was submitted for the IPA analysis. However, from the total of 241 genes, only 193 were considered for further analysis, as the rest represented unmapped or duplicates genes. For the analysis, the log2 ratio cutoff was set to 1. Only genes and direct relationships were included. The preeminent canonical pathways comprised of oxidative phosphorylation, mitochondrial dysfunction, acute phase response signaling, farnesoid X receptor/retinoid X receptor (FXR/RXR) activation, and protein ubiquitination pathways. Its corresponding p-values and ratios could be seen in Table 4. The top diseases and disorder cluster notably included skeletal and muscular disorders and metabolic diseases; despite those pathways ranked third and fourth, we consider them relevant to this study, such as molecular and cellular functions; nucleic acid metabolism; small molecule biochemistry; DNA replication, recombination and repair; energy production; and cell-to-cell signaling and interaction, integrated the classification. Additional information about the top networks, top lists and top molecules were also obtained from the IPA analysis. Detailed information could be seen in Supplementary Table S3.

For a more specific inquiry, we performed individual runs employing subsets of genes according to its expression levels. The array of analyzed under-expressed genes comprises 42 entities and the correspondent top canonical pathways were represented by FXR/RXR activation, acute phase response signaling, LXR/RXR activation, complement system and arginine degradation I. Contrarily, the over-expressed genes list included 151 elements, showing oxidative phosphorylation, mitochondrial dysfunction, protein ubiquitination, telomere extension by telomerase, and phospholipase C signaling as the highest ranked biopathways (Supplementary Table S4).

### Mitochondrial protein-coding genes

**Figure 9:**
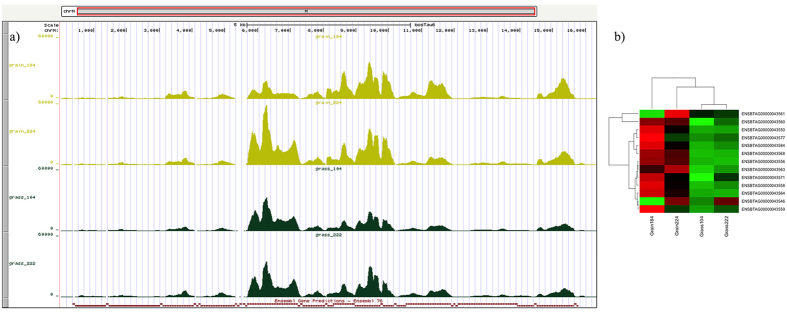
Bovine mitochondrial coding genes. (**a**) Peaks obtained from the alignment of reads to the mitochondrial genomic reference during RNA-Seq analysis; yellow and green peaks correspond to grain and grass-fed samples respectively. (**b**) Heatmap of the 13 mitochondrial protein-coding genes according to their expression levels. The dendogram demostrates that grass-fed animals have a more consistent mitochondrial gene expression profile than grain-fed individuals.

The bovine genome includes 13 mitochondrial protein-coding genes. Considering previous reports that mentioned the essential role of mitochondria in muscle cells and our result from the enrichment analysis, we decided to evaluate the gene expression levels for all of them from the RNA-Seq data and then perform a cluster analysis. Figure 9a depicts distribution of reads from RNA-Seq analysis across the mitochondrial chromosome; it clearly shows the different gene expression profiles between grass and grain-fed individuals. Additionally, Fig. 9a allows visualizing grossly the location and magnitude of peaks, suggesting which genes can be more relevant for each condition as well. We found that most genes increased their expression levels in grain-fed animals compared to grass-fed animals. Although the dendrogram in Fig. 9b, which shows the expression level of the 13-mitochondrial genes, indicates more similarity between grass-fed samples, the resulting heatmap demonstrates a perfect association of the individuals regarding their type of diet.

### Analysis for verification of the RNA-Seq results

**Figure 10:**
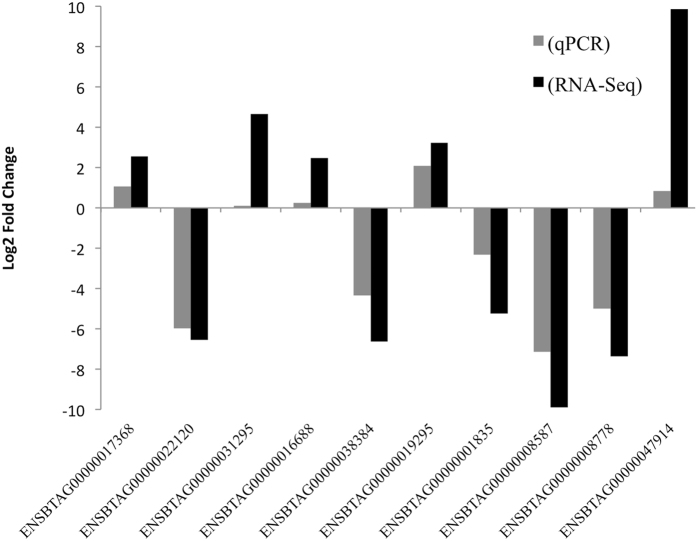
Validation of the RNA-Seq result by qPCR. The y-axis represents the log2 fold change of gene expression; x-axis shows the Ensembl names of genes employed for validation. Black and gray bars depict the RNA-Seq and qPCR results, respectively.

q-PCR analysis has been performed in 10 significant DE genes. Figure 10 demonstrates the certainty of our method, which detect the right direction of the gene expression change in 100% of the genes. The gray bars represent the log2 Fold change values from qPCR and the black bars the corresponding value detected within the RNA-Seq analysis. It is also noticeable the unsteady values reported by the q-PCR and RNA-Seq approaches. However, the q-PCR approach always yielded lower expression and the difference from RNA-Seq approach was quite stable across samples. Genes ENSBTAG00000031295, ENSBTAG00000016688 and ENSBTAG00000047914 presented the largest discrepancy between methods; meanwhile gene ENSBTAG00000022120 exhibited parallel result in both techniques.

### Integrated pathway-level analysis

The result comprises 2,196 canonical pathways from 11 databases (Supplementary Dataset 4). Each row represents a pathway that not only has independent p and q values for genes and metabolites; but also joint p and q values, which makes the result more comprehensible. A systematic way to link analyses from different functional levels always has been problematic. The applied method is just one of the proposed approaches that fit well for our purposes. Unsurprisingly, metabolism was the number one pathway in the list with a joint q-value of 3.81^−30^. The citric acid cycle (TCA), and respiratory electron transport also had significant q-value of 3.95^−15^. According to the KEGG database, oxidative phosphorylation appears first, including 16 genes and 3 metabolites with a joint q-value of 5.11^−11^. The same bio-pathway also arises first in the IPA analysis. Protein digestion and absorption, purine metabolism, amino acyl-tRNA biosynthesis, vitamin digestion and absorption, biosynthesis of unsaturated fatty acids, fat digestion and absorption, pentose phosphate pathway, arginine and proline metabolism, mineral absorption, citrate cycle (TCA cycle), and taste transduction are the significant KEGG pathways (q < 0.05) that included both, genes and metabolites respectively. We chose the KEGG database for our results because it describes pathways more specifically and more detailed than other databases, which frequently define pathways with too broad barriers that are complex to interpret biologically.

## Discussion

Our studies demonstrate that growth rate, gene expression, and metabolite levels in LD muscle are different in response to the grass-fed and grain-fed regimens, indicating that the diet-type influences gene expression and other physiological pathways, thus modulating the host metabolism to regulate its own meat composition and quality. Additionally, previous studies in our laboratory have identified a large series of candidate genes that showed such differences in expression related to beef tenderness^23^,^24^. Taking all of the results together, we can explore the metabolomic profiles in grass-fed and grain-fed beef. The major objective of this project is to determine if these differences are real, and to discover more related effects.

### Polyunsaturated fatty acids

It is well known that muscle from grain-fed animals contains a higher percentage of total fat than that of grass-fed animals, and that the location (inter- or intramuscular) and composition of this fraction could influence both meat tenderness and quality^25^,^26^. Our results summarized in Fig. 4 also confirm the lower total lipid concentration in muscle obtained from grass fed individuals. However, muscle from grass-fed cattle possessed elevated levels of the n3 lipids eicosapentaenoate (EPA), docosapentaenoate (DPA), and docosahexaenoate (DHA). Conversely, n6 polyunsaturated fatty acids (PUFAs) such as dihomo-linolenate, arachidonate, and docosapentaenoate (DPA n6) were diminished in these animals. Our results are in agreement with published studies demonstrating muscle from grass-fed animals has an enrichment of n3 to n6 PUFAs^27^,^28^. The omega3/omega6 ratio has been linked to metabolic perturbations like type 2 diabetes, adipose and liver inflammation. Omega-6 and omega-3 promote and reduce inflammation respectively^29^,^30^. Omega-3 EPA, DPA, and DHA were also 5 to 10 fold higher in the blood of grass-fed steers compared to grain-fed animals which may reflect dietary intake. Although a modest elevation in a portion of n6 fatty acids was observed in the blood of grass-fed cattle, these observations could reflect recent feeding compared to grain counterparts prior to sacrifice as suggested by 6 to 22 fold elevations in multiple food components including homostachydrine and stachydrine found in plants, such as alfalfa, and higher levels of bile acids, such as glycocholate and glycochenodeoxycholate in the muscle and blood. Notably, lower levels of n6 PUFAs such as arachidonate in the muscle tissue of grass-fed animals may suggest a decreased capacity for the generation of inflammatory lipid mediators. The arachidonic acid, precursor of the pro-inflammatory eicosanoids, is released from membrane phospholipids during inflammatory activation to produced prostaglandins and leukotrienes^31^. Dietary supplementation with long chain omega-3 fatty acids could exert protective effects in various inflammatory disorders. During inflammation, the secreted EPA competes with arachidonic acid for enzymatic metabolism, resulting in less synthesis of inflammatory and chemotactic derivatives. Furthermore, the endocannabinoids oleic ethanolamide and palmitoyl ethanolamide were reduced in muscle from grass-fed animals. Oleic and palmitoyl ethanolamide cannot stimulate cannabinoid receptors, but can stimulate PPAR-alpha activity and have been shown to be elevated in diabetic obese patients^32^, suggesting a potentially similar metabolic disruption in grain finished cattle. PPAR-alpha is a ligand-activated transcription factor with a main regulation effect of lipids metabolism in the liver. PPAR activation produces up-regulation of genes implicated in transport of fatty acids, fatty binding and activation, and peroxisomal and mitochondrial fatty acid beta-oxidation^33^.

### Lipid oxidation

Interestingly, medium and long chain fatty acids including pelargonate, palmitate, and stearate were elevated in grass-fed blood samples, but modestly reduced in related muscle tissue. Differences in fatty acid levels between these matrices may reflect alterations in lipid metabolism or recent feeding as described earlier that may impact the lipid signature. Diminished fatty acid abundance in muscle in grass-fed cattle was in agreement with evidence in the literature demonstrating that this meat exhibits less marbling compared to grain finished counterparts^34^. Similarly, lower levels of myristate and palmitate were reported in grass- versus grain-fed meat^6^,^35^. Differences in fatty acid levels may indicate a change in complex lipid hydrolysis considering lower levels of glycerol and several monoacylglycerols including 2-oleoylglycerol in the blood and muscle of grass-fed cattle suggest decreased triglyceride availability and/or degradation compared to grain counterparts. Additionally, carnitine conjugated fatty acids such as stearoylcarnitine and oleoylcarnitine were reduced in muscle tissue, but accumulated in the blood of grain-fed animals and may be indicative of a change in fatty acid β-oxidation. Specifically, higher levels of carnitine conjugated fatty acids in the blood of grain-fed cattle may suggest inefficient fatty acid oxidation as suggested by the depletion of free carnitine. Furthermore, modestly higher levels of the dicarboxylic fatty acids azelate and undecanedioate in muscle from grain-fed individuals may reflect altered β-oxidation. Dicarboxylic fatty acids are generated when the terminal methyl group of a fatty acid is converted into a carboxyl group and are often indicative of ω-oxidation. Although the catabolism of fatty acids typically occurs via β-oxidation in the peroxisomes and/or mitochondria, fatty acid ω-oxidation occurs in the smooth endoplasmic reticulum and often serves as a rescue pathway for conditions where β-oxidation is disrupted or overwhelmed. Interestingly, inefficient lipid oxidation in grain-fed animals may still be higher than grass-fed counterparts as suggested by modestly elevated levels of the ketone body 3-hydroxybutyrate (often generated in the presence of excess acetyl-CoA and historically a marker of lipid oxidation in datasets) in the blood and muscle. Furthermore, the grain diet may be ketogenic. Together, these observations suggest complex lipid hydrolysis and lipid availability may be greater in muscle from grain-fed diet and result in elevated lipid oxidation that may be partially disrupted. In contrast, lipid accumulation in the blood of grass-fed animals may suggest dietary feeding decreased incorporation into triglycerides and decreased absorption by tissues.

### Glucose metabolism

Compared to grass-fed animals, animals finished on a grain diet exhibited considerable higher levels of blood glucose. This finding may reflect a difference in dietary intake or decreased uptake of glucose by tissues. Similarly, significantly higher levels of sorbitol pathway metabolites fructose and sorbitol, often generated by the reduction of glucose excess in muscle from grain-fed diet, may be indicative of increased glucose availability to the tissue. In humans, it has been reported that the increased activity of the sorbitol pathway of glucose metabolism is one of the leading factors in the pathogenesis of diabetic complications^36^. The negative effects of increasing the sorbitol pathway activity under hyperglycemic conditions comprise intracellular sorbitol accumulation with an increment in osmotic stress and production of fructose, which is more potent than glucose to generate non-enzymatic glycosylation^37^. Controversially, other groups have reported that increased activity of the sorbitol pathway could be beneficial rather than detrimental because the pathway detoxifies toxic lipid peroxidation products. In contrast, elevated levels of maltopentaose, maltotetraose, maltotriose and maltose in muscle of grass-fed individuals may indicate glycogen degradation in order to maintain glucose homeostasis. Differences in glycogen availability may be of interest in future studies considering that glycogen in post-mortem muscle can be converted to lactate, causing the meat to become more acidic and less predisposed to browning. A study in lambs demonstrated that lairage time exerts a slight effect on carcass quality traits, affecting most of the meat quality parameters at 24 h post-mortem. Additionally, the glycogen content in liver and longissimus dorsi muscle decreased as lairaged time increased (0 to 12 hours). However, the muscle from lambs that were sacrificed after 3 hours of lairage had the highest pH and shear force measures^38^. Grain-fed cattle may also exhibit enhanced glycolysis as evidenced by the accumulation of pyruvate and lactate in the blood as well as increased glucose utilization for anabolic growth as suggested by the accumulation of the pentose phosphate pathway (PPP) metabolites ribose 5-phosphate and the isobar for pentulose 5-phosphate in the muscle. The pentose phosphate pathway is a biochemical process paralleling to glycolysis that produces NADPH and 5-carbon sugars; its main role is anabolic rather than catabolic, thus its metabolites of higher concentration observed in grain fed animals coincided with their metabolic stage. Furthermore, PPP facilitates NADPH regeneration necessary for glutathione reduction, nucleotide synthesis and anabolic growth. Indeed, reduced levels of NADPH in the muscle of grain-fed animals may suggest utilization for biogenesis and therefore may require recycling via glucose shuttling.

### Cortisol level and animal well-being

Theoretically, changes in glucose homeostasis may be mediated by cortisol, which is a naturally occurring glucocorticoid produced by the adrenal glands^39^,^40^,^41^. Related to metabolism, cortisol assists in the maintenance of blood glucose level through the stimulation of gluconeogenesis in the liver, from non-carbohydrate substrates (amino acids, glycerol, lactate and propionate)^42^. Additionally, cortisol inhibits the glucose uptake in muscle and adipose tissue, and stimulates the fat breakdown in the last type of tissue to released glycerol, one of the substrates used for gluconeogenesis. Thus, higher cortisol levels in grain-fed blood suggest that grain feeding might increase glucose availability for uptake by muscle tissue and subsequently promote utilization for glycolytic metabolism and anabolic growth^43^,^44^,^45^. Intriguingly, cortisol has been recognized as the “stress hormone” for its higher levels during the “fight or flight” response to stress^46^,^47^,^48^. Thus, plasma cortisol level has been used to assess the stress in different species^49^,^50^,^51^,^52^. In humans, stress-induced hyperglycemia is commonly observed in patients admitted to the intensive care unit (ICU), even when the blood glucose level has been previously normal^53^. The elevation of cortisol could result from the activation of the hypothalamic-pituitary-adrenal axis. Cortisol increased 4 fold immediately after extenuating exercise in humans, returning to the baseline within 17 hours^26^,^54^. Housing system constitutes an important factor in animal welfare; measurement of total protein, lysozyme, cortisol, serum and feces corticosterone concentration and GR-α gene expression demonstrated that tie-stall causes more stress than loose system in cows^55^. Plasmocortisol levels in dogs was used to determine the level of stress during confinement in public shelters; the results suggest that confinement induces a prolonged activation of the hypothalamic-pituitary-adrenal axis with possible implications for welfare of confined dogs^49^. From an economic prospective, stress can negatively influence meat quality^56^. However, no difference in plasma cortisol concentration associated with feeding system was observed after transportation and immediately before slaughter in lambs^56^. Recently, it has been reported that hair cortisol could be useful for measuring chronic stress in dairy cattle^57^. As we have known, stress is associated with increased levels of circulating corticosteroids, mainly cortisol (corticosterone)^58^,^59^. Theoretically, there are two biopathways associated with initial stress response to environmental stimuli, the sympathetic-adrenal-medullary (SAM) axis and the hypothalamic-pituitary-adrenal (HPA) axis^60^, respectively. Activation of the HPA axis results in a significant release of the steroid hormone from the cortex of the adrenal glands. Systemic cortisol concentrations increase immediately after threat and last for a long time and also recur if the threat is still present^61^. Physiologically, cortisol has multiple effects on organ systems involved in glucose homeostasis, blood pressure regulation, behavior modification, inflammation, and immune function. Corticosteroids can bind to the glucocorticoid receptors in the cytoplasm, and then the complex is transferred to the nucleus, acting as transcription factors to modulate gene expression down-stream^62^,^63^,^64^. Since the binding sites are ubiquitous throughout the genome; corticosteroid activation during stress could cause wide modifications in gene expression^65^,^66^. Considering that both groups of animals have been treated under similar conditions during loading, transport and termination, the observed difference in cortisol level could be generated by a combination of factors present in each type of feeding system. Grain-fed animals received high-energy diet in confinement, predisposing them to suffer more metabolic disorders or infectious diseases than their grass-fed counterpart. Additionally, grain-fed animals were maintained in groups and fed *ad libitum*, therefore the competition among individuals could become another source of stress. Consequently, long-term exposure to higher cortisol level may cause immune depression, suggesting that confined animals fed with grain diets are more prone to infections and diseases, a relevant matter for animal welfare and public health as well^67^,^68^. Interestingly, Japanese Wagyu cattle breeders feed their animals with enormous amount of grain twice daily from 11 months of age until slaughter (28 to 30 months of age). From 11 to 18 months, the concentrate content of diet increases from approximately 37 to 87% with a concomitant decrease of roughage^69^; nevertheless the animals appear to be perfectly healthy and calm. The cattle are only eating, seating or sleeping, and they look fine. However, the natural behavior of ruminants is to graze, consume grass freely and ruminate on the pasture, following its essential instinct. Thus, instead of considering cortisol as a marker of “pure stress” we conceive that cortisol level could be indicative for abnormal feeding or adverse environmental circumstances for cattle against instinctive feeding. Therefore, the higher cortisol level in the blood of the grain-fed group observed in our study strongly suggests that grass-fed animals generally suffered less adverse circumstances. We think grass-fed animals mainly benefit from moving freely, exercising their muscles and expressing innate behavior in a more natural habitat.

### Gene expression analysis and functional annotation

RNA-Seq and posterior functional analyses broadly concurred with the findings revealed by the metabolomics data. IPA reported oxidative phosphorylation as the top pathway. Oxidative phosphorylation occurs in the mitochondria of cells, which use their structure and enzymes to produce ATP. During this process electrons from donors are transferred to acceptors generating energy, which is used to form ATP. Mitochondrial dysfunction was the next pathway according to IPA; it arises when the reactive oxygen species (ROS)-mediated oxidative stress overcomes the antioxidant defense system. Metabolic fluctuations can trigger oxidative stress and cause mitochondrial dysfunction. Unexpectedly, the pathways oxidative phosphorylation, mitochondrial dysfunction, and protein ubiquitination, included among the top five significant, only comprised up-regulated genes in the grass-fed group, suggesting that these biological processes are sped up in the grass-fed animals.

The most represented cellular compartment was certainly the mitochondria. The 13 genes of the mitochondria genome are potential biological markers for diet type in beef. We found nucleic acid metabolism; small molecule biochemistry; DNA replication, recombination and repair, energy production; and cell-to cell signaling interaction as top molecular and cellular functions, which are primarily associated with the metabolites divergences observed in the two groups. The metabolomic and transcriptomic analyses lead to similar results making our findings very reliable and suggesting that both approaches could be combined for more solid conclusions. Thus, we implemented the integrated pathway-level analysis, which resulted in oxidative phosphorylation as the most significance pathway. Certainly, 16 genes and 3 metabolites assigned to this pathway demonstrated notable divergences between diet regimens. However, to identify which are more critical for altering the content and quality of the beef needs more advanced genome editing methods in the future.

In Summary, our results provide evidence that grass-fed animals produce tender beef with lower total fat, higher omega3/omega6 ratio and superior protein content than grain-fed animals, which are beneficial for the health of consumers. Finally, the higher blood cortisol level in the grain-fed group strongly suggests that grass-fed animals experience less stress; mainly from moving freely, exercising their muscles and expressing usual species behavior in a more natural environment.

## Materials and Methods

### Animals

The steers came from a closed Wye Angus herd with very similar genetics. The grass-fed group was comprised of steers that received alfalfa and orchard grass hay, clover and orchard grass pasture, or orchard grass and alfalfa pasture. The grass-fed individuals consumed grazed alfalfa upon availability and bales during winter, so they were not exposed to any corn, any form of grain, or any form of feed by-products. The alfalfa and grass hay were harvested from land that has had minimal fertilizer and no application of pesticides or inorganic chemicals. The control group was fed a conventional diet consisting of corn silage, soybean, shelled corn and minerals. The pastures were managed without fertilizers, pesticides, or any chemical additives. In order to demonstrate the nutritional differences between diets, a sample from each regimen was collected in two consecutive years. The analytical results summarized in columns for both diets (Supplementary Table S2) reflect the averaged value from those two years estimated for each individual parameter. When the animals reached the required market weight, they have been accordingly prepared, weighted and shipped for termination. To avoid any extrinsic variation during all these processes–from the previous day at the farm to the moment of sacrifice–, both groups were treated similarly. Animals were shipped late afternoon (around 5:00 pm) the date before and were fasted but with free access to water until the moment of termination. We considered that season of slaughter could influence some results–especially the functional–, but we reproduced the time frame that grass-fed animals need to achieve the weight determined by the market. At the slaughter plant, 10 ml whole blood sample from the jugular vein was collected in EDTA tubes and directly stored at −80 °C. Blood collection for both groups were performed immediately before slaughtering; the environmental condition and time of collection were similar for the two groups (approximately between 6:30–9:30 am). Immediately after termination, a small piece of *longissimus dorsi* muscle was obtained from each hot carcass at the level of the 12^th^ intercostal space and immediately frozen in dry ice for posterior analysis. Commercially, the *longissimus dorsi* muscle is highly valuable and constitutes a reference for beef quality studies, allowing reasonably comparison of different results among them. All animal experiments were conducted following NIH guidelines for housing and care of laboratory animals and in accordance with The University of Maryland at College Park (UMCP) regulations after review and approval by the UMCP Institutional Animal Care and Use Committee (permit number R-08-62).

### Warner-Bratzler Shear Force

Immediately after slaughter, carcasses were deposited and maintained in 4 °C chambers for maturation during 14 days. Then, steaks of one inch (2.54 cm) from the longissimus dorsi muscle were cut at the 12^th^ intercostal space, vacuum packaged and immediately frozen at −20 °C. For the Warner-Bratzler Shear Force assessment, the steaks were firstly thaw at 4 °C and then cooked using a George Foreman grill to a core temperature of 70 °C. To measure the internal temperature of the steaks, an Oakton digital thermometer (Temp JKT Acorn series) was employed. After the samples cooled down to room temperature, six cores of tissue of 1.27 cm in diameter and parallel to the fibers were obtained employing a hand-held coring device. The Warner–Bratzler shear force (WBSF) was determined for each core utilizing an Instron #5442 Test Machine (Norwood, MA). The final WBSF value was calculated averaging the measurements of six cores from the same steak.

### Metabolomic and Metabolite analyses

#### Samples description and preparation

Global biochemical profiles were determined from whole blood and muscle tissue in eight individuals from each group: the grain-fed group displayed a younger age (range 443–490 days) than the grass-fed group (range 619–667 days). Metabolomic profiling analysis was performed by Metabolon as previously described^70^. Each sample received was accessioned into the Metabolon LIMS system and was assigned a unique identifier by the LIMS that was associated with the original source identifier only. All samples were maintained at −80 °C until processed. Samples were prepared using the automated MicroLab STAR^®^ system from Hamilton Company. A recovery standard was added prior to the first step in the extraction process for QC purposes. Sample preparation was conducted using aqueous methanol extraction process to remove the protein fraction while allowing for maximum recovery of small molecules. The resulting extract was divided into four fractions: one for analysis by UPLC/MS/MS (positive mode), one for UPLC/MS/MS (negative mode), one for GC/MS, and one for backup. Samples were placed briefly on a TurboVap^®^ (Zymark) to remove the organic solvent. Each sample was then frozen and dried under vacuum. Samples were then prepared for the appropriate instrument, either UPLC/MS/MS or GC/MS.

#### Ultra-high Performance Liquid Chromatography/Mass Spectroscopy (UPLC/MS/MS)

The LC/MS portion of the platform was based on a Waters ACQUITY ultra-performance liquid chromatography (UPLC) and a Thermo-Finnigan linear trap quadrupole (LTQ) mass spectrometer, which consisted of an electrospray ionization (ESI) source and linear ion-trap (LIT) mass analyzer. The sample extract was dried and then reconstituted in acidic or basic LC-compatible solvents, each of which contained 8 or more injection standards at fixed concentrations to ensure injection and chromatographic consistency. One aliquot was analyzed using acidic positive ion optimized conditions and the other using basic negative ion optimized conditions in two independent injections using separate dedicated columns. Extracts reconstituted in acidic conditions were gradient eluted using water and methanol containing 0.1% formic acid, while the basic extracts, which also used water/methanol, contained 6.5 mM ammonium bicarbonate. The MS analysis alternated between MS and data-dependent MS^2^ scans using dynamic exclusion. Raw data files were archived and extracted as described below.

#### Gas Chromatography/Mass Spectroscopy (GC/MS)

The samples destined for GC/MS analysis were re-dried under vacuum desiccation for a minimum of 24 hours prior to being derivatized under dried nitrogen using bistrimethyl-silyl-triflouroacetamide (BSTFA). The GC column was 5% phenyl and the temperature ramped from 40° to 300 °C in a 16 minute period. Samples were analyzed on a Thermo-Finnigan Trace DSQ fast-scanning single-quadrupole mass spectrometer using electron impact ionization. The instrument was tuned and calibrated for mass resolution and mass accuracy on a daily basis. The information output from the raw data files was automatically extracted as discussed below.

#### Quality Assurance/Quality Control

For QA/QC purposes, additional samples were included with each day’s analysis. These samples included extracts of a pool of well-characterized human plasma, extracts of a pool created from a small aliquot of the experimental samples, and process blanks. QC samples were spaced evenly among the injections and all experimental samples were randomly distributed throughout the run. A selection of QC compounds was added to every sample for chromatographic alignment, including those under test. These compounds were carefully chosen so as not to interfere with the measurement of the endogenous compounds.

#### Data extraction and compound identification

Raw data was extracted, peak-identified and QC processed using Metabolon’s hardware and software. Compounds were identified by comparisons to library entries of purified standards or recurrent unknown entities. Metabolon maintains a library based on authenticated standards that contain the retention time/index (RI), mass to charge ratio (m/z), and chromatographic data (including MS/MS spectral data) on all molecules present in the library. Furthermore, biochemical identifications are based on three criteria: retention index within a narrow RI window of the proposed identification, nominal mass match to the library +/− 0.2 amu, and the MS/MS forward and reverse scores between the experimental data and authentic standards. The MS/MS scores are based on a comparison of the ions present in the experimental spectrum to the ions present in the library spectrum. While there may be similarities between these molecules based on one of these factors, the use of all three data points can distinguish and differentiate biochemicals. More than 3,500 commercially available purified standard compounds have been acquired and registered into LIMS for distribution to both the LC and GC platforms for determination of their analytical characteristics. Poly unsaturated fatty acids mentioned in Table 1 were identified from beef samples following this approach.

#### Statistical Analysis

Missing values (if any) were assumed to be below the level of detection. However, biochemicals that were detected in all samples from one or more groups but not in samples from other groups were assumed to be near the lower limit of detection in the groups in which they were not detected. In this case, the lowest detected level of these biochemicals was imputed for samples in which that biochemical was not found. Following log transformation and imputation with minimum observed values for each compound, Welch’s two-sample t-test was used to identify biochemicals that differed significantly between experimental groups. Pathways were assigned for each metabolite, allowing examination of overrepresented pathways.

### Gene Expression Analysis

#### mRNA Extraction and RNA-Seq Library Preparation

The mRNA was extracted directly from muscle samples employing the Oligotex Direct mRNA Mini Kit (Qiagen, Valencia, CA) according to the manufacturer’s protocol. For synthesis of the first cDNA, SuperScript II Reverse Transcriptase Kit (Invitrogen, Carlsbad, CA) with random hexamers (Invitrogen, Carlsbad, CA) was applied in a final volume of 40 μl. A combination of Polymerase I (NEB, Ipswich, MA), 2 U of RNase H (Invitrogen) and 30 nmol of deoxyribonucleotides, followed by a 2.5 hour of incubation at 25 °C, was used to generated the second cDNA strand. For an optimal quality, QIAquick PCR purification kit (Qiagen) was employed to enrich the PCR products. Index libraries were assembled utilizing Multiplex Sample preparation Oligonucleotide Kit (Illumina, San Diego, CA) complying with the provided instructions. After, 50 μl of the purified samples was placed in a Bioruptor sonicator (Diagenode, Denville, NJ) for 40 min, obtaining double cDNA fragments of approximately 200 to 500 bp. The yielding fragments were repaired employing the end repair module (NEB) while the subsequent 3′A addition was achieved by Klenow Fragment (NEB). T4 DNA ligase (NEB) generated the ligation of the Solexa adaptors (Illumina) to the double-stranded cDNA fragments. The amplification step comprised the use of index and adaptor primers during 18 PCR cycles. The resulted PCR products with a length of 200 to 500 bp were filtrated by QIAquick Gel purification kit (Qiagen). Finally, clustering and sequencing was performed on the Hi-Seq 2000 (Illumina) platform.

#### RNA-Seq Data Analysis

An ultrafast, memory efficient short read aligner called Bowtie has been employed to align the sequenced reads to the BosTau6 (Bos_Taurus_UMD_3.1) reference genome, which has been downloaded from the UCSC Genome Bioinformatics site (http://genome.ucsc.edu)^71^. Data quality assurance, data manipulation and file format conversion have been achieved through the implementation of different programs: SAMtools, bedtools, HTSeq and the R based packages, ShortRead, Rsamtools and Biostrings^72^,^73^,^74^. The GenomicRanges and edgeR programs were employed for the statistical analysis (Bioconductor, http://www.bioconductor.org)^75^,^76^. GenomicRanges reported the reads counts and edgeR performed the gene expression analysis, fitting a negative binomial distribution model and then applying a generalized linear model (GLM) likelihood ratio test. In this approach, the common, trended and tagwise variations were estimated, increasing the sensitivity of the method to detect genuine and minimal differences, this facilitates posterior functional and pathway analyses. Additionally, 13 mitochondrial protein-coding genes were extracted from the RNA-Seq result to undergo a clustering analysis. This step was supported by previous outcomes from functional examinations that pointed out the importance of mitochondria activity in muscle^77^,^78^.

#### RNA-seq validation

qPCR was employed for result verification (iCycler iQ PCR system, Bio-Rad, Hercules, CA). The qPCR reactions were performed in a final volume of 20 ul, employing the QuantiTect SYBR Green PCR Kit (Qiagen) following the manufacturer’s instructions. For primer design, the NCBI tool (http://www.ncbi.nlm.nih.gov/tools/primer-blast/index.cgi) was used considering that all amplicons would embody at least one intron. Supplementary Dataset S1 shows the primers sequences.

#### Pathway Analysis

Differentially expressed gene functions were annotated using the Ingenuity Pathway Analysis (IPA) tool (http://www.ingenuity.com/products/ipa). The program inquires the IPA Knowledge database for information and direct relationships of genes and endogenous chemicals. This creates algorithmically generated networks and clustering of the data into biological functions and diseases that are overrepresented in the scrutinized data. Resulting networks are created *de novo* based upon input genes, proteins or chemicals. The program also determines the high representation of signaling and biological pathways. The method employs the Fisher’s exact test, determining the proportion of genes mapped to a function or pathway in the sample and then compares it to the ratio in the reference set. Statistical similarity means no biological effect. The result consisted of functional networks, disease and disorders, molecular and cellular functions, physiological system development and function, top canonical pathways, upstream regulators, and top toxicity lists.

#### Integrated pathway-level analysis of transcriptomic and metabolomics data

The web-based Integrated Molecular Pathway-Level Analysis (IMPaLA) (http://impala.molgen.mpg.de) was employed for the joint pathway analysis of gene expression and metabolite concentration; it performed an over-representation analysis with the provided lists of genes and metabolites employing 11 databases, which included more than 3000 pre-annotated pathways^79^. The results list the pathways that may be altered at the transcriptional level, the metabolomic level, or both. This approach provides combined evidence of pathway deregulation and permits to identify biological pathways that would not have been distinguished by independent single functional level analyses. For this case, all entities present in the pathways were used as background lists. Then, a hypergeometric test determined the significance of each pathway based on its overlap with the uploaded lists. The result reports pathways that include at least one gene or metabolite from the input arrays. The table comprises of pathway name, source, size, and overlap with the provided entities, and p-values obtained with the appropriate statistical test for each pathway. The joint p-values have been calculated following a previously described method^80^. To control for multiple comparisons, the false discovery rate approach was applied, assigning a q-value for every listed pathway^81^. By clicking on the pathway name, the user is directed to the source database and a summary for the correspondent pathway is displayed.

## Additional Information

**How to cite this article**: Carrillo, J.#;#000E.9; A. *et al*. Integrated metabolomic and transcriptome analyses reveal finishing forage affects metabolic pathways related to beef quality and animal welfare. *Sci. Rep.* **6**, 25948; doi: 10.1038/srep25948 (2016).

## Supplementary Material

Supplementary Information

Supplementary Dataset S1

Supplementary Dataset S2

Supplementary Dataset S3

Supplementary Dataset S4

## Figures and Tables

**Table 1:** Polyunsaturated Fatty acids in beef.

**Biochemical**	Mean Grain*	Mean Grass*	**Fold Change**	**p-value**	**q-value**
eicosapentaenoate (EPA; 20:5n3)	0.6103	1.9478	0.31	0.0000	0.0001
docosapentaenoate (n3 DPA; 22:5n3)	0.7342	1.3533	0.54	0.0034	0.0043
docosahexaenoate (DHA; 22:6n3)	0.7733	1.3363	0.58	0.0013	0.0023
linoleate (18:2n6)	1.0001	0.8906	1.12	0.5070	0.1831
linolenate [alpha or gamma; (18:3n3 or 6)]	0.4920	2.0852	0.24	0.0000	0.0000
dihomo-linolenate (20:3n3 or n6)	1.2911	0.7161	1.80	0.0050	0.0055
arachidonate (20:4n6)	1.5782	0.7931	1.99	0.0095	0.0092
adrenate (22:4n6)	1.0393	0.9055	1.15	0.3768	0.1484
docosapentaenoate (n6 DPA; 22:5n6)	2.2328	0.5677	3.93	0.0001	0.0004
dihomo-linoleate (20:2n6)	1.2098	0.9403	1.29	0.6033	0.2092
mead acid (20:3n9)	1.7035	0.8769	1.94	0.0541	0.0343

^*^Units of measure correspond to relative mass spectrometry intensities measured in equivalent amounts of muscle tissue.

**Table 2:**
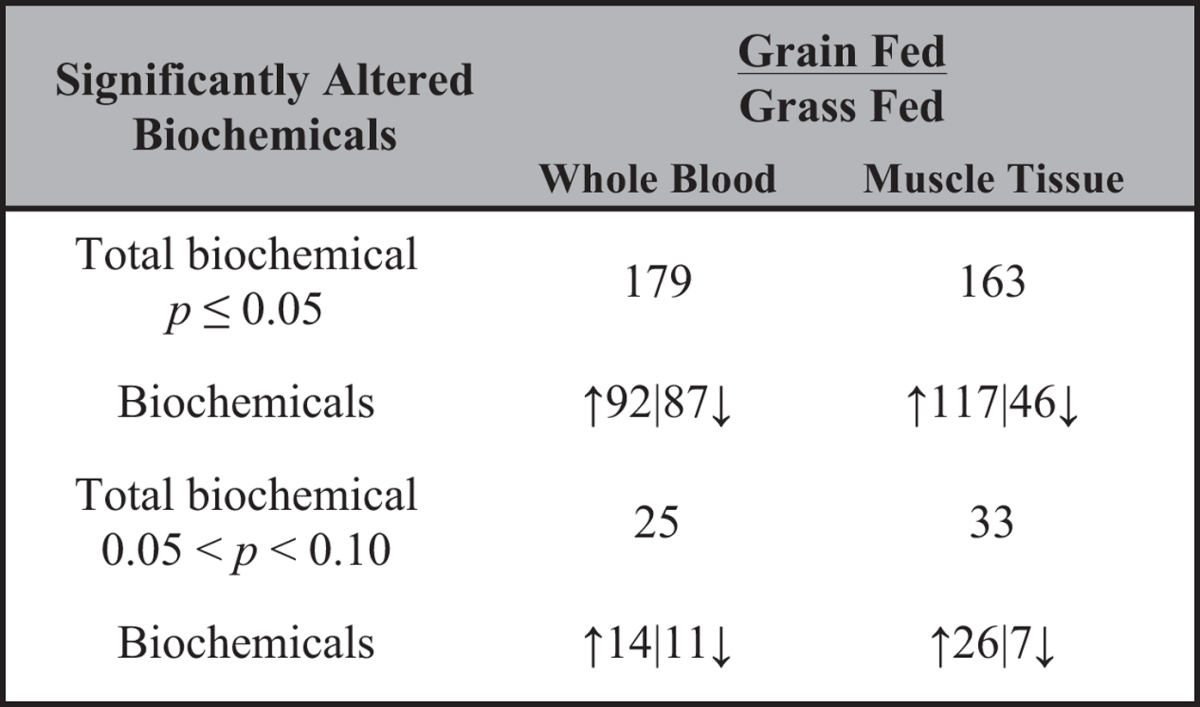
Summary of identified biochemicals according to the Welch’s test.

Arrows indicate up- and down-regulation of the compounds in grass-fed individuals.

**Table 3:** Sample descriptors for RNA-Seq analysis.

**Sample**	**Reads Processed**	**Aligned Reads**	**Aligned (%)**
Grain_184	15,260,337	11,288,315	73.97
Grain_224	17,141,418	12,349,227	72.04
Grass_104	15,158,827	11,532,709	76.08
Grass_222	15,269,225	11,634,528	76.20

**Table 4:** Top canonical pathways from IPA analysis.

**Name**	**p-value**	**Ratio**
Oxidative Phosphorylation	2.14e-11	13/109 (0.119)
Mitochondrial Dysfunction	6.03e-09	13/171 (0.076)
Acute Phase Response Signaling	1.65e-04	8/169 (0.047)
FXR/RXR Activation	1.67e-04	7/127 (0.055)
Protein Ubiquitination Pathway	2.43e-03	8/255 (0.031)
