# Supplementary material for: Integrated metabolomic and transcriptome analyses reveal finishing forage affects metabolic pathways related to beef quality and animal welfare
Source: Sci Rep. 2016 May 17;6:25948. doi: 10.1038/srep25948 (PMC4869019; doi:10.1038/srep25948)
Supplement: Supplementary Information [file srep25948-s1.pdf]

Integrated metabolomic and transcriptome analyses reveal finishing forage affects metabolic pathways related to beef quality and animal welfare

José A. Carrillo[1], Yanghua He[1], Yaokun Li[2], Jianan Liu[1], Richard A. Erdman[1], Tad Sonstegard[3], Jiuzhou Song[1*]

[1]Department of Animal & Avian Sciences, University of Maryland, College Park, MD 20742, USA
[2]College of Animal Science and Technology, Northwest A&F University, Yangling, Shaanxi, P.R. China, 712100
[3]Animal Genomics and Improvement Laboratory, Agricultural Research Service, United States Department of Agriculture, USA
[*]Corresponding author: songj88@umd.edu

Supplementary Table S1 Clinical Blood Test Parameters. Averaged values and standard errors calculated from 8 individuals in each feeding system.

Test	Grain	Grass	Unit	p-value
White Blood Cells	11.13±2.13	8.35±4.59	$x10^3$ cells/uL	0.25
Neutrophils	3.23±1.60	2.48±0.50	$x10^3$ cells/uL	0.34
Lymphocytes	6.68±1.52	6.61±1.42	$x10^3$ cells/uL	0.94
Monocytes	0.54±0.19	0.49±0.10	$x10^3$ cells/uL	0.63
Eosinophils	0.47±0.50	0.45±0.21	$x10^3$ cells/uL	0.92
Basophils	0.11±0.03	0.08±0.02	$x10^3$ cells/uL	0.06
Large Unstained Cells	0.08±0.06	0.03±0.02	$x10^3$ cells/uL	0.11
% Neutrophils	28.60±9.67	24.60±3.95	%	0.41
% Lymphocytes	60.36±10.16	65.00±5.16	%	0.38
% Monocytes	4.84±1.11	4.90±0.92	%	0.92
% Eosinophils	4.42±4.51	4.34±1.83	%	0.97
% Basophils	1.06±0.42	0.67±0.38	%	0.16
% Large Unstained Cells	0.72±0.40	0.36±0.17	%	0.09
Red Blood Cells	8.19±0.50	8.29±0.37	$x10^6$ cells/uL	0.72
Hemoglobin	12.34±1.72	12.58±1.00	g/dL	0.79
Hematocrit	34.38±4.71	34.84±2.63	%	0.85
Mean Corpuscular Volume	41.88±4.26	42.02±2.75	fL	0.95
Mean Corpuscular Hemoglobin	15.04±1.54	15.20±1.06	pg	0.85
Mean Corpuscular Hemoglobin Concentration	35.92±0.32	36.14±0.23	g/dL	0.24
Red Cell Distribution Width	18.62±0.67	19.44±0.89	%	0.13
Plateles	510.60±134.34	384.20±187.41	$x10^3$ cells/uL	0.25
Mean Platelet Volume	5.94±0.27	6.98±2.36	fL	0.35
CO2	21.94±2.79	25.38±1.69	mmol/L	0.04
Glucose	101.60±15.21	85.00±3.87	mg/dL	0.04

Supplementary Table S2 Feed Components Analysis

Components	Dry matter basis (%)	
	Grain Diet	Grass Diet
Crude protein	13.9	17.5
Adjusted crude protein	13.9	17.5
Soluble Protein	47	28
Acid Detergent Fiber	18.4	42.4
Neutral Detergent Fiber	29.7	56.6
Lignin	2.6	7.1
NFC	47.1	18.4
Starch	35.6	0.2
Crude fat	2.9	2.5
Ash	6.47	10.12
TDN	73	60
NEL, Mcal/Lb	0.77	0.56
NEM, Mcal/Lb	0.79	0.56
NEG, Mcal/Lb	0.51	0.3

Supplementary Table S3 Ingenuity Pathway Analysis of all differentially expressed genes

Top Diseases and Bio Functions

Diseases and Disorders

Name	p-value	# Molecules
Neurological Disease	4.28E-06 - 1.66E-02	53
Psychological Disorders	4.28E-06 - 1.55E-02	40
Auditory Disease	4.97E-06 - 1.70E-02	8
Skeletal and Muscular Disorders	1.29E-05 - 1.21E-02	47
Metabolic Disease	2.74E-05 - 1.55E-02	46

Molecular and Cellular Functions

Name	p-value	# Molecules
Nucleic Acid Metabolism	1.15E-06 - 1.41E-02	20
Small Molecule Biochemistry	1.15E-06 - 1.60E-02	47
DNA Replication, Recombination, and Repair	2.30E-06 - 9.13E-03	12
Energy Production	2.30E-06 - 9.14E-03	14
Cell-To-Cell Signaling and Interaction	8.29E-05 - 1.70E-02	22

Physiological System Development and Function

Name	p-value	# Molecules
Organ Morphology	1.62E-05 - 1.63E-02	23
Skeletal and Muscular System Development and Function	1.62E-05 - 1.41E-02	13
Hematological System Development and Function	8.29E-05 - 1.70E-02	34
Immune Cell Trafficking	8.29E-05 - 1.70E-02	25
Humoral Immune Response	4.63E-04 - 2.86E-03	4

Top Canonical Pathways

Name	p-value	Ratio
Oxidative Phosphorylation	2.14E-11	13/109 (0.119)
Mitochondrial Dysfunction	6.03E-09	13/171 (0.076)
Acute Phase Response Signaling	1.65E-04	8/169 (0.047)
FXR/RXR Activation	1.67E-04	7/127 (0.055)
Protein Ubiquitination Pathway	2.43E-03	8/255 (0.031)

Top Networks

ID	Associated Network Functions	Score
1	Nucleic Acid Metabolism, Small Molecule Biochemistry, DNA Replication, Recombination, and Repair	51
2	Cell-mediated Immune Response, Cellular Development, Cellular Function and Maintenance	48
3	Lipid Metabolism, Molecular Transport, Small Molecule Biochemistry	35
4	Cellular Assembly and Organization, Cellular Function and Maintenance, Hereditary Disorder	26
5	Connective Tissue Disorders, Inflammatory Disease, Skeletal and Muscular Disorders	26

Top Tox Lists

Name	p-value	Ratio
Mitochondrial Dysfunction	7.44E-09	13/174 (0.075)
Renal Safety Biomarker Panel (PSTC)	1.47E-05	3/6 (0.5)
FXR/RXR Activation	1.67E-04	7/127 (0.055)
Positive Acute Phase Response Proteins	2.53E-03	3/30 (0.1)
Glutathione Depletion - CYP Induction and Reactive Metabolites	5.15E-03	2/12 (0.167)

Top Molecules

Log Ratio up-regulated

Molecules	Exp. Value	Exp. Chart
RBP2	↑10.995	
CCL5	↑10.706	
VIL1	↑10.500	
FCGBP	↑10.255	
ALPI	↑9.773	
FABP2	↑9.727	
CDHR2	↑9.473	
GIMAP7	↑9.413	
KRT20	↑9.156	
LCK	↑9.025	

Log Ratio down-regulated

Molecules	Exp. Value	Exp. Chart
SERPINA3*	↓-10.589	
KRT5	↓-10.266	
TTR	↓-10.201	
CAMK1D	↓-10.143	
A2ML1	↓-10.000	
PRSS35	↓-9.980	
HELQ	↓-9.710	
CA12	↓-9.616	
TLR9	↓-9.613	
PLA2G2A	↓-9.552	

Supplementary Table S4 Ingenuity Pathway Analysis according to direction of gene expression in grass-fed individuals

Down-regulated genes<br>Top Canonical Pathways		
Name	p-value	Ratio
FXR/RXR Activation	2.05E-07	6/127 (0.047)
Acute Phase Response Signaling	1.1E-06	6/169 (0.036)
LXR/RXR Activation	1.05E-04	4/121 (0.033)
Complement System	2.05E-03	2/33 (0.061)
Arginine Degradation I (Arginase Pathway)	8.11E-03	1/4 (0.25)

Up-regulated genes<br>Top Canonical Pathways		
Name	p-value	Ratio
Oxidative Phosphorylation	8.45E-13	13/109 (0.119)
Mitochondrial Dysfunction	2.68E-10	13/171 (0.076)
Protein Ubiquitination Pathway	4.79E-04	8/255 (0.031)
Telomere Extension by Telomerase	4.94E-03	2/15 (0.133)
Phospholipase C Signaling	7.23E-03	6/239 (0.025)
